# The Influence of Auditory Dysfunction on Ergonomic Workplace Design for Aging Employees

**DOI:** 10.3390/audiolres15050109

**Published:** 2025-08-29

**Authors:** Matjaž Maletič, Albin Kotnik, Zvone Balantič

**Affiliations:** 1 Faculty of Organizational Sciences, University of Maribor, 2000 Maribor, Slovenia; zvone.balantic@um.si; 2 AUDIO BM d.o.o., Italijanska Ulica 8, 1000 Ljubljana, Slovenia; a.kotnik@audiobm.si

**Keywords:** hearing dysfunction, older workers, noise exposure, ergonomics, audiometric assessments, workers’ perception

## Abstract

**Background/Objectives**: This research focuses on the increasing presence of older workers in the labor market, a group particularly vulnerable to hearing problems due to age-related changes and prolonged noise exposure. **Methods**: The research combines theoretical and empirical approaches to investigate the impact of noise on the workplaces of older employees. The empirical component is based on two primary methods: a survey and audiometric testing to assess participants’ hearing abilities. The study included a sample of 50 older workers, all with diagnosed hearing loss. **Results**: The results of the survey showed that most older workers are regularly exposed to noise at work, which has long-term negative effects on their hearing. This highlights the need to introduce appropriate protective measures such as personal protective equipment, insulation of noise sources, and raising awareness about the dangers of noise. In addition to the questionnaire survey, the analysis of hearing measurements revealed that all respondents had significant bilateral hearing loss, with sensorineural hearing loss being the most prevalent type. **Conclusions**: This study highlights the negative impact of chronic noise exposure in the workplace on the hearing, communication and productivity of older workers and emphasizes the importance of combining preventive measures, hearing protection and workplace adaptations to promote their well-being and performance.

## 1. Introduction

In today’s society, living conditions are constantly improving, partly due to increased awareness of healthier lifestyles. One of the main reasons for the increase in life expectancy is the significant advances in the field of medicine. When these factors are considered together with declining birth rates and migration trends, it becomes clear that the average age of the European population is steadily increasing [1].

Population aging is a global trend that has led to a steady increase in the number of older workers on the labor market [2]. This demographic shift brings with it particular challenges, as aging is often accompanied by natural physiological changes, one of the most common of which is hearing loss [3]. Hearing plays a crucial role in ensuring quality of life, effective communication and social inclusion for older workers. Importantly, hearing loss not only affects individuals, but also has far-reaching implications for organizations and society as a whole [4]. People with hearing loss often tend to withdraw socially, which can lead to isolation and depression. Research has shown that “noise exposure associated with advancing age can lead to a deterioration in quality of life by impairing communication, social interactions and mental health” [5]. In response to increasing scientific knowledge about the health risks associated with noise exposure [6] and the introduction of stricter legal standards [7], considerable progress has been made in noise reduction and noise protection systems in recent years [8]. However, despite these developments, noise exposure remains an important issue for older workers, who may be more susceptible to the negative effects of noise.

With increasing age, hearing naturally declines due to degenerative changes in the auditory system. With increasing age, the prevalence of chronic diseases such as diabetes and high blood pressure also increases, which can further favor the deterioration of hearing. It is well known that hearing is affected by cumulative noise exposure over time—a factor that becomes increasingly important as we undergo aging. As noted in previous studies (e.g., [9]), age-related hearing loss (ARHL) and chronic disease often form a negative cycle in which deteriorating hearing and poor health are closely linked [10]. Based on a bibliometric analysis of ARHL and the identification of citation bursts, the growing importance of and research interest in ARHL-related studies in recent years can be clearly demonstrated [11].

As the population ages, it is increasingly important to understand the needs and challenges of older workers to ensure sustainable and inclusive employment practices [12]. Previous research has examined the impact of noise exposure in the workplace from the perspective of older workers, highlighting the negative effects on hearing health, communication skills and overall work performance [13]. Despite the growing awareness of the effects of occupational noise on older workers, most existing studies tend to examine either the physiological effects of noise exposure through audiometric testing [14] or the subjective experiences of workers through surveys [15]—rarely both. This discrepancy limits a holistic understanding of how measurable hearing impairment relates to perceived workplace support and ergonomic adjustments. Furthermore, the literature often overlooks how older workers themselves perceive the appropriateness of noise reduction strategies and hearing-related adjustments in their work environment. To address this gap, the present study combines objective hearing tests with survey data on workers’ perceptions of workplace ergonomics and hearing protection. This combined approach enables a more comprehensive assessment of the interactions between noise exposure, hearing loss and perceived organizational support that affect older workers’ well-being and work performance. The study therefore fills a critical gap in the literature by examining how older workers evaluate the relevance and appropriateness of noise reduction strategies and hearing-related adjustments in their work environment. This dual-focus approach contributes new insights into the design of age-appropriate and ergonomically supportive workplaces.

The remainder of this paper is organized as follows. Section 2 describes the materials and methods and explains the mixed-methods approach used in the study, which combined a structured survey with audiometric assessments of respondents’ hearing. Section 3 presents the key findings, which include both the quantitative hearing data and the participants’ perceptions of ergonomic measures in the workplace. Section 4 discusses the findings in relation to existing research, emphasizing the implications for hearing preservation and ergonomic adaptation in the aging workforce. Finally, Section 5 concludes the paper with a summary of key findings, methodological limitations and recommendations for future research and practice.

## 2. Materials and Methods

This study combines theoretical and empirical approaches to investigate the effects of noise on the workplaces of older workers. In the theoretical part, data were collected using descriptive and analytical methods, drawing on existing literature on topics such as older workers, hearing, hearing aids and working conditions in noisy environments.

The empirical part of the study is based on two key methods: a survey and the measurement of the respondents’ hearing ability. The survey was conducted both physically and online and was completed by 50 older workers diagnosed with hearing loss. For the purposes of this study, an older worker is defined as a person over the age of 50, in line with the definitions commonly used in the existing literature [16]. A purposive sampling strategy was used to recruit participants who met the following criteria: aged 50 years or older, currently employed or in the early stages of retirement, and formally diagnosed with hearing loss by a certified otorhinolaryngologist. Participants were selected from the client base of AUDIO BM d.o.o. and invited to participate either online or in person. Although the sample was not randomly selected, the targeted approach ensured the inclusion of individuals directly relevant to the research objectives. A 100% response rate was achieved, and all participants provided informed consent. Data was collected using a structured questionnaire and internal data analysis via AUDIO BM d.o.o.’s NOAH software system (version 4.12.1). The majority of participants were aged 61–65 years (40%), followed by those aged 56–60 years (36%) and 50–55 years (24%). The sample consisted of 60% male and 40% female respondents (Table 1). The online survey was conducted using 1KA—One Click Survey (https://www.1ka.si/d/en; accessed between 19 January 2024 and 14 February 2024), while some older participants completed it in person at the AUDIO BM branch in Kranj. Prior to participation, all respondents were informed by the research team, who explained the purpose of the study and obtained informed consent for the use of their audiograms (hearing measurements) for research purposes. The structured questionnaire used in the study comprised a total of 32 questions, focusing on self-reported hearing loss, occupational noise exposure, and the respondents’ perceptions of workplace hearing protection and communication challenges. The questionnaire also included general demographic items such as age and gender. For the purposes of this analysis, 15 of the 32 questions were selected based on their relevance to the study objectives. Some variables were used in their original form, while others were recoded or aggregated to simplify interpretation. The answers to the questionnaire were directly linked to the audiometric data and personal information of the participants, allowing an integrated analysis of subjective and objective indicators.

The study participants selected were older workers with hearing loss whose diagnosis had been confirmed by an ENT specialist. We analyzed audiometric data collected by healthcare professionals and used encrypted respondent identifiers to link the data while ensuring privacy and confidentiality.

Hearing loss was quantified using the Fowler method, a standardized approach that estimates the percentage of hearing impairment based on pure-tone audiometry thresholds at key speech frequencies [17]. Specifically, the Fowler–Sabine method—also known as the American Medical Association (AMA) method—was employed [18,19]. This method calculates a frequency-weighted average of hearing thresholds at 500 Hz (15%), 1000 Hz (30%), 2000 Hz (40%), and 4000 Hz (15%). Binaural hearing loss (%HL, Fowler) was subsequently determined using the following formula:

$$Binaural\;Loss\;\left(\%\right)=\;\frac{\left(7\;\times Better\;Ear\;\%\right)+(1\;\times Worse\;Ear\;\%)}{8}$$

This methodology aligns with established clinical and medico-legal standards for assessing functional hearing impairment in Slovenia.

## 3. Results

The analysis of the hearing measurements, as shown in Table 2, revealed a pronounced bilateral hearing loss in all participants. The data clearly indicate that sensorineural hearing loss predominates, which is consistent with its status as the most common form of hearing impairment. This type of hearing loss is usually caused by damage to the inner ear or auditory nerve and is often irreversible. The uniformity of findings in the sample suggests a systemic or age-related etiology, which warrants further investigation into possible causes such as noise exposure, genetic predisposition or ototoxic medication. However, this article focuses primarily on the ergonomic factors that can contribute to or exacerbate hearing loss and emphasizes the need for comprehensive prevention strategies in the workplace and environmental settings.

**Figure 1:**
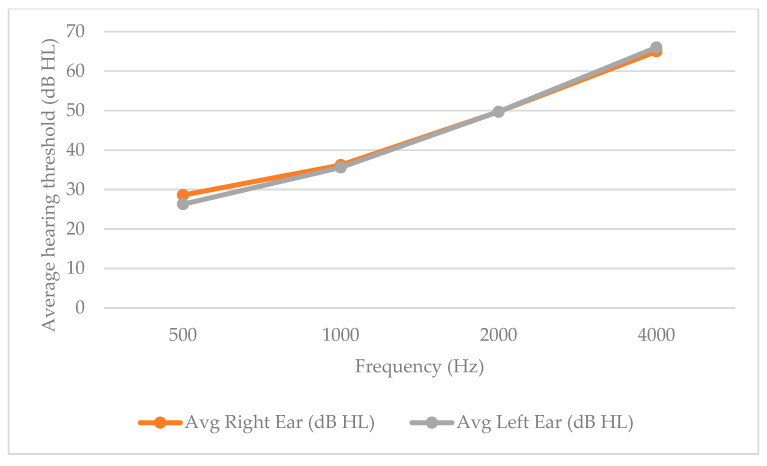
Average hearing thresholds (in dB HL) across speech-relevant frequencies (500 Hz, 1000 Hz, 2000 Hz, and 4000 Hz) for the right and left ears of all respondents (*n* = 50).

Furthermore, Figure 1 illustrates the average pure-tone hearing thresholds for the right and left ear at the most important speech frequencies (500, 1000, 2000, and 4000 Hz). The data reveal a gradual increase in hearing thresholds at higher frequencies, which is consistent with typical age-related hearing loss patterns observed in the study population. Both ears demonstrate similar audiometric profiles.

The hearing threshold data suggest the presence of different patterns of hearing impairment among the respondents, possibly indicating different underlying pathological mechanisms. However, without bone conduction measurements, a specific classification into sensorineural, conductive or mixed types of hearing loss cannot be definitively confirmed. We calculated the average hearing loss at speech frequencies that are critical for the perception of spoken language and environmental sounds. A slight asymmetry was found between the left and right ear, with the left ear having an average hearing loss 2 decibels (dB) higher than the right ear. Although this difference is relatively small, it can still affect sound perception and communication in daily life [20].

In addition, we calculated the degree of hearing loss using the Fowler method, which is based on the results of pure-tone audiometry at speech frequencies and provides a quantitative assessment of hearing loss in percent. The average hearing loss of the respondents was 38.4 dB, which provides a comprehensive insight into the extent of hearing impairment. The highest hearing loss measured using the Fowler method was 91.10% and the lowest was 10.80%, illustrating the variability of hearing loss within the study population. Calculating hearing loss according to the Fowler method enables people who fulfill certain criteria to receive aids such as vibrating alarm clocks, induction loops and telephones for the hearing-impaired.

Table 3 (right ear) and Table 4 (left ear) show the results of the independent-samples *t*-test, which was performed to test whether there are statistically significant differences in the mean values between two independent groups. The respondents were divided into two groups based on the median of the hearing loss calculated using the Fowler method. The analysis shows that at a significance level of 5 (*p* > 0.05), the null hypothesis (H_0_) cannot be rejected. This means that there is no statistical evidence of significant differences between the mean values of the two groups.

Our study found that 34% of respondents had been working in noisy environments for less than 20 years, while 66% had been working in such environments for 20 years or more. An independent *t*-test was performed to investigate whether mean %HL (Fowler) scores differed according to duration of noise exposure (Table 5). The results showed no statistically significant difference in hearing loss between those who had worked in noisy environments for less than 20 years and those who had been exposed for 20 or more years.

In order to determine the proportion of respondents who answered “yes” to the question of interest (e.g., Do you think your hearing has deteriorated since you started working in the company where you have spent most of your career?), a binomial test with a test quota of 0.50 was carried out (Table 6). Of the respondents, 66% (*n* = 27) answered “yes” (Group 1), while 34% (*n* = 14) answered “no” (Group 2). The result of the two-sided binomial test was *p* = 0.060. Although a larger proportion of respondents answered “yes”, the difference from the expected 50% was not statistically significant at the usual alpha level of 0.05. This suggests that although there is a tendency for more positive responses, we cannot say with certainty that the proportion is significantly different from chance. However, the result is marginal and could indicate a trend that should be investigated further in future studies.

Furthermore, a total of 53.7% of respondents stated that they were rarely or occasionally exposed to noise at work, while 46.3% were frequently or very frequently exposed to noise. When analyzing the %HL (Fowler), no statistically significant difference was found between the two groups of respondents, i.e., between those with scores below and those with scores above the reference median (Table 7). These results suggest that the frequency of noise exposure does not lead to a significant difference between those with lower and higher levels of hearing loss as measured by %HL (Fowler). One possible explanation is that factors beyond frequency of exposure—such as individual susceptibility, use of hearing protection, type and intensity of noise, or non-occupational noise exposure—may play a more influential role in the development of hearing loss. As a result, the frequency of exposure alone cannot sufficiently explain the observed differences in hearing outcomes between the two groups.

Furthermore, respondents were asked to rate the noise level at their workplace according to their perception: Group 1 corresponds to those who reported a low noise level, while Group 2 includes those who reported a medium or high noise level. The binomial test was performed to determine whether the proportion of respondents perceiving a low noise level deviated significantly from an expected proportion of 0.50 (Table 8). In Group 1, 28 respondents (56%) reported low noise levels, while in Group 2, 22 respondents (44%) reported medium or high noise levels. The test resulted in a two-sided significance value of *p* = 0.480, which means that there is no statistically significant difference from the expected equal distribution. This indicates that the proportion of respondents who perceive low noise exposure at work is about the same as the proportion of those who perceive medium or high noise exposure based on their subjective experience.

Table 9 shows a cross-tabulation of general satisfaction with the workplace, broken down by the perceived noise level at the workplace. Respondents who perceived the noise level at their workplace to be low (Group 1) were more likely to report that noise had no (10 out of 22) or only a slightly negative impact (7 out of 22) on their job satisfaction. In contrast, respondents who perceived a medium or high noise level (Group 2) were more likely to report a negative impact on satisfaction, with the majority reporting a strongly negative impact (14 out of 28) or a somewhat negative impact (8 out of 28). These results indicate a clear trend: higher perceived noise levels in the workplace are associated with greater negative impacts on overall job satisfaction. Conversely, lower noise levels are associated with a lower perceived impact on satisfaction, suggesting that noise exposure may be an important factor influencing employees’ overall experience at work.

The results of the study provide important insights into the impact of noise on respondents’ ability to communicate with colleagues, superiors and customers within the organization. According to the participants, noise poses a significant challenge to effective communication in the workplace. It interferes with the successful exchange of information and can lead to misinterpretation, delays in task completion and reduced productivity. Understanding these effects is therefore crucial to developing appropriate strategies to manage noise and improve the working environment in organizations.

Table 10 shows the distribution of self-reported work productivity across the different levels of perceived noise exposure in the workplace. Among respondents reporting a low noise level (Group 1), productivity scores are more evenly distributed: 25% (5 out of 20) reported high productivity, 55% (11 out of 20) reported medium productivity and 20% (4 out of 20) reported low productivity. In contrast, those who were confronted with a medium or high noise level (Group 2) were far less likely to report high productivity (only 4% or 1 out of 27), while the majority reported medium productivity (70%, 19 out of 27) or low productivity (26%, 7 out of 27). These results suggest that higher noise exposure at work is associated with lower perceived productivity. Specifically, fewer workers exposed to medium or high levels of noise perceive themselves as highly productive compared to workers in a low-noise environment. This pattern underscores the potentially negative impact of increased noise exposure on work performance and perceived worker productivity.

Our study found that 66% of respondents used a hearing aid during their working life, and 60% of those who used the device said it helped their work in terms of quality and productivity. However, only 48% said they used the device regularly, while the rest only used it occasionally. Furthermore, the majority of respondents (60%) stated that hearing aids improved their work performance, which emphasizes the significant potential of hearing aids to increase occupational efficiency in older workers. However, the results also suggest that hearing aids are not equally effective for everyone. The reasons why some people did not experience an improvement in their work performance through the use of hearing aids may be inadequate fitting of the devices, psychological factors or other individual differences.

The results of the binomial test (Table 11) show that a statistically significantly greater proportion of respondents stated that ergonomic measures to reduce noise-related disturbances at their workplace had not been implemented. Specifically, 73% of participants (*n* = 24) reported that no such measures had been implemented, compared with 27% (*n* = 9) who reported the existence of such measures. The two-sided exact significance value of *p* = 0.014 confirms that this difference is unlikely to be due to chance. These results indicate a notable gap in the implementation of ergonomic adjustments aimed at mitigating noise annoyance in the work environment and highlight an area for organizational improvement.

The results of the binomial test (Table 12) show that a significantly larger proportion of respondents (73%, *n* = 27) stated that their employer actively participates in adapting the working environment, compared to 27% (*n* = 10) who did not perceive such participation. The two-sided exact significance value of *p* = 0.008 confirms that this difference is statistically significant and probably not due to chance. These results suggest that the majority of employees recognize their employer’s active role in changing the workplace, which may have a positive impact on the company’s commitment to addressing employees’ needs and improving working conditions. Respondents were asked about the impact of ergonomic adjustments to reduce noise-related disturbances on their job satisfaction and productivity. Most indicated that these changes had a positive impact on their overall satisfaction and job performance. Participants also mentioned specific changes the company had made, including installation of soundproofing panels, purchase of newer machines, noise-reducing devices on the equipment, installation of new (soundproof) windows, and provision of personal hearing protection devices.

Table 13 shows the results of an independent *t*-test comparing respondents’ ratings of their employer’s noise abatement efforts in the workplace on a scale from 1 (very poor) to 5 (very good). Group 1 consists of people with %HL values (Fowler) below the reference median, while Group 2 includes those with scores above the median. The average rating of employer support was 3.16 (SD = 0.99) in Group 1 and 3.76 (SD = 1.09) in Group 2. The difference between the groups was statistically significant (t (48) = 2.04, *p* = 0.047), indicating that respondents with higher hearing loss scores tended to rate their employer’s attention to noise adaptation more positively than respondents with lower hearing loss scores. Additionally, a Pearson correlation analysis was conducted to examine the relationship between the %HL (Fowler) scores and respondents’ perceptions of their employer’s efforts to adapt to workplace noise, measured on a 5-point Likert scale (1 = very negative to 5 = very positive). The results indicated a significant positive correlation (r = 0.299, *p* = 0.035), suggesting that respondents with greater hearing loss tended to rate employer adaptation efforts more positively. This result could indicate that employees with more severe hearing loss are more aware of, or more sensitive to, their employers’ efforts to address noise-related problems in the workplace. However, it could also indicate that workplaces with employees who have a higher hearing loss implement more visible noise adaptation measures.

It is worth highlighting that 20% of respondents stated that their employer does not participate in adapting the work environment for older workers, suggesting that there may be a need for greater awareness of adapting the workplace to an aging workforce. We also asked older employees whether they had ever requested an adjustment to their job or working hours during the course of their career for age- or health-related reasons. Overall, the results show that while the majority of respondents (84%) have never asked for such adjustments, there is also a proportion of respondents who have been faced with the need for changes to their work environment or work schedule. This shows the importance of adapting workplaces and working conditions to the needs of older workers in order to maintain productivity and well-being in the workplace.

To assess awareness and training regarding workplace safety and the risks of noise exposure, respondents were asked if they had ever attended a lecture or training on workplace safety related to the risks of noise exposure. A binomial test (Table 14) was conducted to determine whether the proportion of “no” responses (Group 1) differed significantly from the expected proportion of 0.50. In this group (*n* = 50), 54% of respondents answered “no”,” but this difference was not statistically significant (*p* = 0.672, two-tailed). This suggests that the proportion of employees who have not participated in such training is not significantly different from chance and that participation in training is roughly evenly distributed—46% said “yes” (Group 2). Although the results are not statistically significant, they do show that less than half of respondents have received noise exposure training, indicating a potential gap in workplace safety training that may warrant further attention.

Furthermore, 82.6% of those who attended a lecture or training course on safety at work in relation to noise exposure stated that they had become more aware of hearing protection in the workplace as a result of the training.

## 4. Discussion

Human factors related to ergonomics and aging include a variety of abilities, e.g., perceptual abilities such as touch, hearing and vision, processes such as perception, memory and judgment, and advanced abilities such as spatial reasoning, decision-making and problem-solving [21]. As the proportion of older workers continues to increase, it is essential that organizations prioritize effective ergonomic practices to ensure a safe and productive work environment. Occupational noise-induced hearing loss—especially unprotected exposure to noise levels above 85 dBA [22]—can be an additional problem for workers over the age of 60. This risk is exacerbated by the natural progression of presbycusis and the cumulative effects of prolonged exposure to noise over the course of a working life [23]. The limited evidence on safety practices and health risks among workers over the age of 60 is a cause for concern [24]. In fact, the relationship between hearing loss in adulthood and employment is still poorly understood [25]. Objective measures such as audiometric testing provide important insights into the extent of hearing impairment, but do not fully capture the life experiences of those affected. It is therefore equally important to consider employees’ perceptions, including how they experience and manage hearing problems in the workplace. Understanding both the subjective and objective dimensions of hearing loss can provide a broader perspective on the impact and enable the development of more effective support strategies and interventions in the workplace. This study contributes to closing this gap by combining theoretical analysis with empirical research focusing on older workers with diagnosed hearing loss. Using a survey and audiometric testing of 50 individuals, the study provides evidence that chronic noise exposure in the workplace has measurable, long-term effects on hearing, particularly on sensorineural hearing loss. The results underline the urgent need for targeted preventive measures, including the use of personal protective equipment, isolation of noise sources and greater awareness among employees and employers. By highlighting the direct impact of noise on communication and productivity, this research underlines the importance of adapting workplaces to support the health and well-being of an aging workforce. In fact, the results of our study indicate that noise poses a significant challenge to effective communication in companies. It has a negative impact on the successful exchange of information, which can lead to misinterpretation, delays in task completion and lower productivity. Therefore, understanding these effects is crucial for developing appropriate strategies to manage noise and improve the working environment in organizations.

Overall, no significant differences were found in the perception of the work environment and the impact of hearing loss between respondents rated above the median and below the median based on the %HL indicator (Fowler). This may indicate that the subjective experience of hearing problems is not necessarily directly proportional to the objectively measured degree of hearing loss. It is possible that individuals develop different coping strategies or have a different sensitivity threshold to the impact of hearing loss in the workplace. These findings emphasize the importance of including both objective measurements and subjective perceptions in a comprehensive assessment of the impact of hearing loss on employee performance and well-being. As highlighted in previous studies [26,27], personal factors can significantly influence the effectiveness of hearing loss prevention strategies and programs. In particular, the use of hearing protection is strongly influenced by perceived self-efficacy, the degree of noise annoyance experienced, perceived barriers and benefits associated with the use of hearing protection, and perceived sensitivity to hearing loss [26]. Studies such as the World Report on Hearing (WRH) [4] have long highlighted the harmful effects of noise on hearing and psychosocial well-being. Our research confirms that noise is not only a direct cause of hearing deterioration, but also has a negative impact on workers’ quality of life and productivity.

Our study found that less than 50% of respondents had attended training on workplace safety in relation to noise exposure, highlighting the need to raise awareness in this area. A study by Svensson et al. [28] found that the vast majority of workers were aware of the risks of loud noise—95% recognized that noise can damage hearing, 90% saw hearing loss as a serious problem and 85% believed that hearing protection devices are effective—but actual use of hearing protection devices remained inconsistent, with significantly fewer workers reporting using them regularly when exposed to noise. Understanding these differences can serve as a basis for designing programs that are tailored to specific groups of workers and can help improve the work environment and productivity in the workplace. Just as important as training employees is the provision of age-specific training for employers aimed at improving their understanding of age-related issues and supporting the development of appropriate policies and strategies [29]. However, not all employers are willing to invest in such training as some perceive a limited return on investment and doubt that they will be able to recoup the costs involved [30].

Furthermore, older workers with more pronounced hearing loss did not report a significantly more negative perception of workplace factors and practices that affect hearing than their peers. However, a limitation of this study is the lack of a control group consisting of respondents without objectively measured hearing impairments. The inclusion of such a comparison group could have provided valuable insights into how hearing status influences the perception of occupational noise and hearing-related measures in the workplace. Future research involving both affected and unaffected workers would improve understanding of the subjective experience of hearing loss and enable the development of more targeted and effective interventions. Despite the above arguments, it could be argued that there are potential differences between objective hearing measurements and the subjective perception of respondents that can be explained by several interrelated mechanisms. First, people often develop cognitive adaptations and compensatory strategies [31], such as increased attention or avoidance of noisy environments, which may make them less aware of difficulties despite measurable hearing loss. In addition, psychosocial factors—such as stress, social support and the stigmatization of hearing loss—significantly influence subjective perceptions and may contribute to individuals experiencing their hearing problems differently [32]. It is also important to emphasize that tolerance to auditory impairment is highly individual; some people perceive difficulties more acutely, while others may not recognize them as significant [33,34]. Finally, while audiometric tests provide accurate and objective data on hearing thresholds, they do not necessarily capture the quality of the hearing experience, such as speech understanding in noisy environments or the perception of auditory distortion, which can contribute to discrepancies between objective and subjective assessments. Building on our previous research [35], this study supports the idea that the inclusion of sound-induced auditory fatigue issues—alongside hearing loss and/or tinnitus—can improve the assessment of hearing-related symptoms in occupational settings where intensive speech communication is required. This more comprehensive approach may provide deeper insights into the impact of demanding acoustic environments on workers’ auditory well-being and communication efficiency.

## 5. Conclusions

This study investigated the effects of workplace noise exposure on older workers, focusing on the impact on well-being, productivity and communication skills. The aim was to gain a comprehensive understanding of how chronic noise exposure in the workplace affects the work experience of older workers in organizations. The results showed that the majority of older workers are regularly exposed to elevated noise levels in the workplace, which has a long-term detrimental effect on their hearing. These findings emphasize the urgent need to take effective preventative measures, including the use of personal protective equipment, acoustic isolation of noise sources and systematic awareness-raising of the risks associated with noise exposure. Employers and organizations should take proactive steps to improve working conditions, in particular by educating their employees about hearing protection and encouraging the consistent use of hearing protection devices. In addition, workplace adjustments—such as reorganizing work tasks or changing workspaces—should be made more frequently to reduce the noise exposure of older workers.

The study also found that the use of hearing aids by older workers significantly improves both communication efficiency and work performance. Hearing aids facilitate interaction with customers, colleagues and superiors, contributing to a more positive and productive working environment. These findings emphasize the need to step up efforts to improve working conditions for older workers. Implementing sustainable and effective hearing protection strategies is crucial to reduce the risk of hearing loss and improve the overall work experience of older workers. A comprehensive and preventative approach is essential to ensure safer and healthier workplaces for older workers.

To summarize, the results indicate that older employees are generally satisfied with their employers’ efforts to reduce noise in the workplace. Nonetheless, it remains crucial to address the impact of noise exposure in the workplace, particularly in the context of an aging workforce. Future research could examine the effectiveness of targeted noise reduction measures for this population, as well as the role of employer attitudes and company policies in shaping noise management practices. In addition, comparative studies between industries or countries with different regulatory frameworks and other circumstances could provide deeper insights into best practices for protecting older workers from noise in the workplace.

## Figures and Tables

**Table 1:** Respondents’ profiles.

Age Category (Years)	*n*	%
50–55	12	24
56–60	18	36
61–65	20	40
Total	50	100
Gender distribution (%)	Male	Female
	60	40

**Table 2:** Analysis of speech frequency measurements within the 500 Hz to 4000 Hz range and corresponding %HL (Fowler) calculations.

Respondent ID	Right Ear (dB HL)				Left Ear (dB HL)				%HL (Fowler)
	500 Hz	1000 Hz	2000 Hz	4000 Hz	500 Hz	1000 Hz	2000 Hz	4000 Hz	
R1	60	70	70	65	70	70	65	80	81.90%
R2	15	30	40	55	10	40	45	55	29.60%
R3	10	15	35	100	10	20	55	115	28.00%
R4	35	55	80	100	25	55	75	100	70.30%
R5	15	15	50	45	15	15	50	40	29.00%
R6	35	35	35	75	30	30	35	55	28.50%
R7	30	45	55	75	30	45	55	70	54.00%
R8	25	35	40	75	20	35	40	85	36.80%
R9	30	35	65	65	25	35	65	65	52.30%
R10	35	45	50	55	30	40	55	60	48.90%
R11	45	40	40	25	50	45	45	35	32.50%
R12	25	35	45	55	30	35	45	55	36.60%
R13	30	40	45	45	25	35	35	40	26.20%
R14	45	50	65	60	25	25	30	40	23.40%
R15	20	35	65	65	20	40	65	60	51.70%
R16	35	20	20	40	25	20	15	45	10.80%
R17	25	45	70	85	20	45	65	110	59.60%
R18	15	15	25	75	10	15	45	70	21.90%
R19	30	35	50	65	40	50	50	75	46.70%
R20	55	60	55	65	60	65	60	65	70.10%
R21	35	40	35	65	55	65	70	80	41.70%
R22	20	35	55	45	15	25	40	50	27.00%
R23	15	50	65	70	20	35	50	80	47.40%
R24	15	25	30	60	20	25	40	70	23.70%
R25	10	10	45	65	10	10	10	60	14.40%
R26	10	10	30	65	10	10	25	55	15.80%
R27	20	25	35	65	15	15	25	95	22.00%
R28	35	45	65	105	15	20	45	95	38.30%
R29	20	35	45	55	10	25	30	50	21.20%
R30	15	20	60	65	5	20	65	65	43.30%
R31	50	50	45	75	45	50	60	55	55.70%
R32	15	30	45	60	20	30	45	55	33.60%
R33	10	30	50	60	15	30	45	50	31.60%
R34	35	30	20	30	35	30	30	30	15.30%
R34	40	70	70	60	45	70	65	65	73.90%
R36	25	45	50	50	30	50	55	50	46.10%
R37	10	20	35	50	10	20	30	40	14.80%
R38	10	20	50	65	10	20	50	75	37.40%
R39	10	10	25	60	10	10	40	60	17.60%
R40	20	35	50	60	30	40	60	65	43.80%
R41	50	40	55	50	10	15	45	40	27.00%
R42	85	90	120	120	65	55	75	90	83.10%
R43	50	50	75	90	15	35	55	90	51.80%
R44	20	25	35	65	20	30	40	65	27.60%
R45	30	50	40	40	20	30	35	45	24.40%
R46	5	5	30	75	15	35	65	70	25.30%
R47	100	100	120	120	40	90	110	120	91.10%
R48	20	20	30	70	90	85	85	90	33.20%
R49	30	35	40	50	25	30	45	55	31.60%
R50	5	5	35	50	20	15	55	70	20.70%
Average	28.60	36.20	49.70	65.00	26.30	35.60	49.70	66.0	38.38%

Note: Hearing thresholds were measured using pure-tone audiometry (PTA) and are expressed in decibels Hearing Level (dB HL). %HL (Fowler) indicates the percentage of binaural hearing loss calculated using the Fowler–Sabine (AMA) method, which uses frequency-weighted thresholds at 500 Hz (15%), 1000 Hz (30%), 2000 Hz (40%), and 4000 Hz (15%).

**Table 3:** Results of the *t*-test for individual frequencies critical for speech comprehension (500 Hz, 1000 Hz, 2000 Hz, and 4000 Hz)—right ear.

Frequency	Symbol	*n*	M	SD	SEM	t (*p*)
500	1	22	25.23	20.67	4.41	−0.45 (0.656)
	2	19	27.63	11.59	2.66	
1000	1	22	33.18	22.86	4.87	−0.64 (0.523)
	2	19	36.84	10.17	2.33	
2000	1	22	50.45	21.65	4.62	0.70 (0.489)
	2	19	46.32	15.08	3.46	
4000	1	22	68.18	18.87	4.02	1.19 (0.242)
	2	19	61.05	19.48	4.47	

Note: *n* represents the sample size, M denotes the arithmetic mean, SD stands for standard deviation, and SEM refers to the standard error of the mean. Symbol 1 indicates a Fowler hearing loss score below the reference median, while symbol 2 denotes a score above the reference median.

**Table 4:** Results of the *t*-test for individual frequencies critical for speech comprehension (500 Hz, 1000 Hz, 2000 Hz, and 4000 Hz)—left ear.

Frequency	Symbol	*n*	M	SD	SEM	t (*p*)
500	1	22	21.82	13.23	2.82	−1.22 (0.229)
	2	19	27.89	18.51	4.25	
1000	1	22	33.64	21.22	4.52	−0.74 (0.461)
	2	19	37.89	14.08	3.23	
2000	1	22	52.05	18.49	3.94	0.49 (0.626)
	2	19	49.47	14.42	3.31	
4000	1	22	68.18	19.91	4.25	0.70 (0.489)
	2	19	63.42	23.69	5.44	

Note: *n* represents the sample size, M denotes the arithmetic mean, SD stands for standard deviation, and SEM refers to the standard error of the mean. Symbol 1 indicates a Fowler hearing loss score below the reference median, while symbol 2 denotes a score above the reference median.

**Table 5:** Independent *t*-test results for %HL (Fowler) by duration of occupational noise exposure.

Independent *t*-Test	Group	*n*	M	SD	SEM	t (*p*)
%HL (Fowler)	1	17	39.23	21.38	5.19	0.22 (0.827)
	2	33	37.95	18.50	3.22	

Note: Response categories: 1 = less than 20 years of working in noisy environments; 2 = more than 20 years of working in noisy environments.

**Table 6:** Binomial test results for self-reported hearing deterioration since employment at primary workplace.

Binomial Test	Group	*n*	Observed Prop.	Test Prop.	Exact Sig. (2-Tailed)
Hearing deterioration	1	27	0.66	0.50	0.060
	2	14	0.34		

Note: Response categories: 1 = yes; 2 = no.

**Table 7:** Independent *t*-test results for mean scores of %HL (Fowler) considering the frequency of noise exposure.

Independent *t*-Test	Group	*n*	M	SD	SEM	t (*p*)
%HL (Fowler)	1	22	38.05	19.97	4.26	0.28 (0.395)
	2	19	36.60	13.25	3.04	

Note: Response categories: 1 = rarely or occasionally exposed to noise at work; 2 = frequently or very frequently exposed to noise.

**Table 8:** Binomial test results for perceived workplace noise levels (low vs. medium/high).

Binomial Test	Group	*n*	Observed Prop.	Test Prop.	Exact Sig. (2-Tailed)
Workplace noise level	1	28	0.56	0.50	0.480
	2	22	0.44		

Note: Response categories: 1 = low level of noise; 2 = medium/high level of noise.

**Table 9:** Cross-tabulation of general workplace satisfaction by perceived occupational noise level.

Noise Level	Strong Negative Impact	A Rather Negative Impact	Mildly Negative Impact	No Impact	Total
Low level (1)	2	3	7	10	22
Medium and high level (2)	14	8	4	2	28
Total	16	11	11	12	50

**Table 10:** Cross-tabulation of self-reported work productivity by occupational noise level.

Noise Level	High Productivity	Medium Productivity	Low Productivity	Total
Low level (1)	5	11	4	20
Medium and high level (2)	1	19	7	27
Total	6	30	11	47

**Table 11:** Binomial test results for perceived implementation of ergonomic measures to reduce noise disturbances at the workplace.

Binomial Test	Group	*n*	Observed Prop.	Test Prop.	Exact Sig. (2-Tailed)
Ergonomic measure implementation	1	24	0.73	0.50	0.014
	2	9	0.27		

Note: Response categories: 1 = ergonomic measures were not implemented; 2 = ergonomic measures were implemented.

**Table 12:** Binomial test results for perceived active adaptation of the work environment by the employer.

Binomial Test	Group	*n*	Observed Prop.	Test Prop.	Exact Sig. (2-Tailed)
Active adaptation	1	27	0.73	0.50	0.008
	2	10	0.27		

Note: Response categories: 1 = yes, 2 = no.

**Table 13:** Independent *t*-test results comparing respondents’ ratings of employer noise adaptation efforts.

Independent *t*-Test	Group	*n*	M	SD	SEM	t (*p*)
Noise adaptation effort	1	25	3.16	0.99	0.20	2.04 (0.047)
	2	25	3.76	1.09	0.22	

Note: Response categories: 1 = %HL (Fowler) score below the reference median; 2 = %HL (Fowler) score above the reference median.

**Table 14:** Results of binomial test on participation in noise-related workplace safety training.

Binomial Test	Group	*n*	Observed Prop.	Test Prop.	Exact Sig. (2-Tailed)
Noise-related training	1	50	0.54	0.50	0.672
	2	50	0.46		

Note: Response categories: 1 = no; 2 = yes. The binomial test was conducted using a test proportion of 0.50 (two-tailed).

## Data Availability

The data on which the results presented in this study are based are not publicly available, but can be obtained from the corresponding author on reasonable request.

## Abbreviations

The following abbreviations are used in this manuscript:

ARHL	Age-related hearing loss
ENT	Otolaryngologist (ear, nose, and throat specialist)
%HL (Fowler)	Percentage hearing loss (Fowler method)
WRH	World Report on Hearing

## References

[1] Galof K., Balantič Z. (2021). Making the Decision to Stay at Home: Developing a Community-Based Care Process Model for Aging in Place. Int. J. Environ. Res. Public Health.

[2] Kühn S., Milasi S., Yoon S. (2018). Population Ageing and Future Labour Market Challenges. World Employ. Soc. Outlook.

[3] Balantič Z., Polajnar A., Jevšnik S. (2016). Ergonomija v Teoriji in Praksi: Znanstvena Monografija [Ergonomics in Theory and Practice: Scientific Monograph].

[4] World Report on Hearing. https://www.who.int/publications/i/item/9789240020481.

[5] Shargorodsky J., Curhan G.C., Farwell W.R. (2010). Prevalence and Characteristics of Tinnitus among US Adults. Am. J. Med..

[6] Themann C.L., Masterson E.A. (2019). Occupational Noise Exposure: A Review of Its Effects, Epidemiology, and Impact with Recommendations for Reducing Its Burden. J. Acoust. Soc. Am..

[7] Zeng A., Huang Y., Xin J., Li J., Qiu W., Zhang M. (2024). Progress and Recommendations of Developing Occupational Exposure Limits for Noise–A Systematic Review. Heliyon.

[8] Arezes P., Pereira G., Kroger M., Sampaio P. (2015). Mitigating the Impact of Occupational Noise Exposure for Elderly Workers: Setting the Functional Requirements for an ANC System. Procedia Manuf..

[9] Li F.-F., Fu Z.-Y., Han K., Liang B.-Y., Han Y.-X., Liu Y.-H., Tong B.-S., Liu Y.-C. (2025). Trends and Driving Factors of Age-Related Hearing Loss and Severity over 30 Years: A Cross-Sectional Study. BMC Geriatr..

[10] Davis A., McMahon C.M., Pichora-Fuller K.M., Russ S., Lin F., Olusanya B.O., Chadha S., Tremblay K.L. (2016). Aging and Hearing Health: The Life-Course Approach. Gerontologist.

[11] Wu Q., Liu M., Ma T., Hu Q., Yuan C., Zhang X., Zhang T. (2024). Research Trends and Hotspot Analysis of Age-Related Hearing Loss: A Bibliometric Analysis from 2019 to 2023. Exp. Gerontol..

[12] Kwan C., Drolet J. (2015). Towards Age-Inclusive Sustainable Development Goals: Exploring the Potential Role and Contributions of Community Development. Community Dev. J..

[13] Gopinath B., McMahon C., Tang D., Burlutsky G., Mitchell P. (2021). Workplace Noise Exposure and the Prevalence and 10-Year Incidence of Age-Related Hearing Loss. PLoS ONE.

[14] Pretzsch A., Seidler A., Hegewald J. (2021). Health Effects of Occupational Noise. Curr. Pollut. Rep..

[15] Lee P.J., Lee B.K., Jeon J.Y., Zhang M., Kang J. (2016). Impact of Noise on Self-Rated Job Satisfaction and Health in Open-Plan Offices: A Structural Equation Modelling Approach. Ergonomics.

[16] Haslam C., Haslam R., Clemes S., Kazi A., Duncan M., Twumasi R., Kerr L. (2012). Working Late: Strategies to Enhance Productive and Healthy Environments for the Older Workforce. Proc. Hum. Factors Ergon. Soc. Annu. Meet..

[17] Fletcher H. (1950). A Method of Calculating Hearing Loss for Speech from an Audiogram. Acta Oto-Laryngol..

[18] On the Definition of Hearing Handicap. https://www.asha.org/policy/RP1981-00022/.

[19] (1942). Tentative Standard Procedure for Evaluating the Percentage Loss of Hearing in Medicolegal Cases. Arch. Otolaryngol..

[20] Avan P., Giraudet F., Büki B. (2015). Importance of Binaural Hearing. Audiol. Neurotol..

[21] Stedmon A.W., Howells H., Wilson J.R., Dianat I. (2012). Ergonomics/Human Factors Needs of an Ageing Workforce in the Manufacturing Sector. Health Promot. Perspect..

[22] Chen K.-H., Su S.-B., Chen K.-T. (2020). An Overview of Occupational Noise-Induced Hearing Loss among Workers: Epidemiology, Pathogenesis, and Preventive Measures. Environ. Health Prev. Med..

[23] Huang Q., Tang J. (2010). Age-Related Hearing loss or Presbycusis. Eur. Arch. Otorhinolaryngol..

[24] Farrow A., Reynolds F. (2012). Health and Safety of the Older Worker. Occup. Med..

[25] Shan A., Ting J.S., Price C., Goman A.M., Willink A., Reed N.S., Nieman C.L. (2020). Hearing Loss and Employment: A Systematic Review of the Association between Hearing Loss and Employment among Adults. J. Laryngol. Otol..

[26] Purdy S.C., Williams W. (2002). Development of the Noise At Work Questionnaire to Assess Perceptions of Noise in the Workplace. J. Occup. Health Safety Aust. N.Z..

[27] Welsh J.F., Purdy S.C. (2001). Management of Age Related Hearing Loss. Australas. J. Ageing.

[28] Svensson E.B., Morata T.C., Nylén P., Krieg E.F., Johnson A.-C. (2004). Beliefs and Attitudes among Swedish Workers Regarding the Risk of Hearing Loss. Int. J. Audiol..

[29] Drake C., Haslam R., Haslam C. (2017). Facilitators and Barriers to the Protection and Promotion of the Health and Safety of Older Workers. Policy Pract. Health Saf..

[30] Johnson R.W., Mermin G.B.T., Resseger M. (2011). Job Demands and Work Ability at Older Ages. J. Aging Soc. Policy.

[31] Powell D.S., Oh E.S., Reed N.S., Lin F.R., Deal J.A. (2022). Hearing Loss and Cognition: What We Know and Where We Need to Go. Front. Aging Neurosci..

[32] Liu C., Mavrommatis M.M., Govindan A., Cosetti M.K. (2025). The Stigma of Hearing Loss: A Scoping Review of the Literature Across Age and Gender. Otolaryngol.–Head Neck Surg..

[33] Henry J.A., Theodoroff S.M., Edmonds C., Martinez I., Myers P.J., Zaugg T.L., Goodworth M.-C. (2022). Sound Tolerance Conditions (Hyperacusis, Misophonia, Noise Sensitivity, and Phonophobia): Definitions and Clinical Management. Am. J. Audiol..

[34] Kim S., Arzac S., Dokic N., Donnelly J., Genser N., Nortwich K., Rooney A. (2025). Individual Noise-Tolerance Profiles and Neural Signal-to-Noise Ratio: Insights into Predicting Speech-in-Noise Performance and Noise-Reduction Outcomes. Audiol. Res..

[35] Fredriksson S., Hammar O., Magnusson L., Kähäri K., Persson Waye K. (2016). Validating Self-Reporting of Hearing-Related Symptoms against Pure-Tone Audiometry, Otoacoustic Emission, and Speech Audiometry. Int. J. Audiol..

